# Role of *MdERF3* and *MdERF118* natural variations in apple flesh firmness/crispness retainability and development of QTL‐based genomics‐assisted prediction

**DOI:** 10.1111/pbi.13527

**Published:** 2021-01-06

**Authors:** Bei Wu, Fei Shen, Xuan Wang, Wen Yan Zheng, Chen Xiao, Yang Deng, Ting Wang, Zhen Yu Huang, Qian Zhou, Yi Wang, Ting Wu, Xue Feng Xu, Zhen Hai Han, Xin Zhong Zhang

**Affiliations:** ^1^ College of Horticulture China Agricultural University Beijing China

**Keywords:** *Malus domestica* Borkh., flesh firmness/crispness retainability, BSA‐seq, genomic selection, ethylene‐responsive factor

## Abstract

Retention of flesh texture attributes during cold storage is critical for the long‐term maintenance of fruit quality. The genetic variations determining flesh firmness and crispness retainability are not well understood. The objectives of this study are to identify gene markers based on quantitative trait loci (QTLs) and to develop genomics‐assisted prediction (GAP) models for apple flesh firmness and crispness retainability. Phenotype data of 2664 hybrids derived from three *Malus domestica* cultivars and a *M. asiatica* cultivar were collected in 2016 and 2017. The phenotype segregated considerably with high broad‐sense heritability of 83.85% and 83.64% for flesh firmness and crispness retainability, respectively. Fifty‐six candidate genes were predicted from the 62 QTLs identified using bulked segregant analysis and RNA‐seq. The genotype effects of the markers designed on each candidate gene were estimated. The genomics‐predicted values were obtained using pyramiding marker genotype effects and overall mean phenotype values. Fivefold cross‐validation revealed that the prediction accuracy was 0.5541 and 0.6018 for retainability of flesh firmness and crispness, respectively. An 8‐bp deletion in the *MdERF3* promoter disrupted MdDOF5.3 binding, reduced *MdERF3* expression, relieved the inhibition on *MdPGLR3, MdPME2,* and *MdACO4* expression, and ultimately decreased flesh firmness and crispness retainability. A 3‐bp deletion in the *MdERF118* promoter decreased its expression by disrupting the binding of MdRAVL1, which increased *MdPGLR3* and *MdACO4* expression and reduced flesh firmness and crispness retainability. These results provide insights regarding the genetic variation network regulating flesh firmness and crispness retainability, and the GAP models can assist in apple breeding.

## Introduction

Fruit softening after ripening is important for plant survival and reproduction. However, optimal flesh firmness and crispness are important fruit quality traits determining consumer choice. The retention of these texture attributes during cold storage is critical for long‐term maintenance of fruit quality (Bonany *et al*., 2014; Grammen *et al*., 2019; Nybom *et al*., 2008; Yue *et al*., 2013). Although many quantitative trait loci (QTL) have been identified and several genes have been reported to be involved in fruit storability, molecular insights regarding fruit firmness and crispness retention are currently limited (Eriksson *et al*., 2004; Janssen *et al*., 2008; Marín‐Rodríguez *et al*., 2003; Rose *et al*., 2002; Segonne *et al*., 2014; Soglio *et al*., 2009).

Fruit firmness and crispness are quantitatively inherited and are genetically controlled by polygenes with minor effects. In apple (*Malus domestica* Borkh.), a number of QTLs for fruit firmness and flesh texture were repeatedly mapped on chromosomes 1, 5, 6, 8, 10, 12, 14, 15, 16 and 17, which explained 10–49% phenotypic variance (Bink *et al*., 2014; García‐Gómez *et al*., 2019; King *et al*., 2001; Longhi *et al*., 2012). A set of diagnostic markers have been converted from QTL‐based markers via candidate gene prediction and variation validation (García‐Gómez *et al*., 2019; Leng *et al*., 2017; Mason *et al*., 2018; Vanderzande *et al*., 2018).

Post‐harvest fruit softening, which counteracts flesh firmness and crispness retention, is a complex physiological process involving the biosynthesis of or response to phytohormones and the degradation of cytoskeletons such as polysaccharide (Marondedze and Thomas, 2012; Xu *et al*., 2018). Ethylene biosynthesis is generally considered a primary factor leading to fruit softening. 1‐Aminocyclopropane‐1‐carboxylic acid (ACC) synthase (*ACS*) and ACC oxidase (*ACO*) are the two well‐studied ethylene production‐related genes. QTL‐based markers on *MdACO1* and *MdACS1* have been shown to be responsible for apple fruit firmness and shelf life (Costa *et al*., 2010; Costa *et al*., 2005a; Harada *et al*., 2000; Oraguzie *et al*., 2007). However, the markers on *MdACS1* and *MdACO1* explained only 8.3–11.4% of phenotype variance, indicating that other genes may contribute to the overall effect of low ethylene production in certain apple lines (Colgan *et al*., 2006; Costa *et al*., 2010).

The rapid increase in internal ethylene concentration triggers the transition from the initial slow softening phase to the rapid softening phase in apple (Johnston *et al*., 2001). A functional single‐nucleotide polymorphism (SNP) marker on polygalacturonase gene *MdPG1* (explains up to 10.7% phenotypic variance) was developed from a QTL for fruit post‐harvest firmness (Costa *et al*., 2010). Flesh firmness loss is usually associated with mealiness. The *MdPG1* also contributed most to apple flesh mealiness (Moriya *et al*., 2017). A significant locus spanning pectin methylesterase (*MdPME)* was associated with apple fruit crispness and mealiness in a genome‐wide association study (GWAS) (Amyotte *et al*., 2017). Apples of cultivar ‘Scifresh’ soften slowly because of the relatively low MdPME and MdPG activities throughout fruit development and ripening (Ng *et al*., 2013, 2014).

In addition to the genes for ethylene biosynthesis and pectin degradation, many transcription factors are involved in fine controlling fruit firmness and crispness. Ethylene response factors, MdERF2 and MdERF3 (XM_008339725, should be *MdERF1A* in NCBI), bind to the *MdACS1* promoter and oppositely regulate its transcription during post‐harvest ripening in apple (Li *et al*., 2016). Nevertheless, the genetic regulatory network mediated by these genes and the relationship between them were not clear.

In the last decade, empirical marker‐assisted selection (MAS) for apple crispness has been practically used in breeding programmes, enabling breeders to design the perfect hybrid cross and to select the best seedlings from large populations (Laurens *et al*., 2018; Lezzoni *et al*., 2016; Peace *et al*., 2019). Breeders have noticed that the efficiency of MAS is often not satisfactory for traits involving many loci with minor effects. Genomic selection (GS) emerged as an innovative paradigm twenty years ago in animal breeding and was soon adopted and rapidly studied in fruit plants (Biscarini *et al*., 2017; Gois *et al*., 2016; Imai *et al*., 2018; Iwata *et al*., 2013, 2016; Kumar *et al*., 2019). In apple, GS or genomic prediction was pioneered by Kumar et al. (2012a, 2012b) and has been used in simulative selections for fruit quality traits, harvest date, and scab resistance (Kumar *et al*., 2012a, 2012b; McClure *et al*., 2018; Muranty *et al*., 2015; Peace *et al*., 2019).

Pure GS estimates additive effects and neglects non‐additive allelic effects and non‐allelic epistasis (Muranty *et al*., 2015). For outcrossing species, these non‐additive effects were, however, important for the improvement of various traits. A hierarchical epistasis of *MdSAUR37*/*MdPP2CH*/*MdALMTII* was identified to control fruit malate content (Jia *et al*., 2018). To link MAS and GS, an additive Bayesian QTL analyses model was developed for apple fruit firmness, the GS accuracy (0.57*–*0.94) was higher than that of genomic best linear unbiased prediction (G‐BLUP) model (0.72–0.77) (Bink *et al*., 2014). These data drew attention to QTL‐based GS, which is sometimes called genomics‐assisted prediction (GAP) (Peace *et al*., 2019). Compared with map‐based QTL mapping and GWAS, bulked segregant analysis via sequencing (BSA‐seq) is considered as a cost‐efficient QTL detection approach and has been effectively used in apple (Shen *et al*., 2019).

The objective of the present study was to develop GAP models for flesh firmness and crispness retainability. Towards this, QTLs were identified from three apple F1 hybrid populations using BSA‐seq approach. To convert significant QTL‐based markers to diagnostic markers, candidate genes were predicted from QTL intervals, assisted by using RNA‐seq and parental re‐sequencing. Then, the allelic variations in candidate genes were validated to confirm the function of the variations. The results can facilitate dissecting the genetic variation network and obtaining functional markers for apple flesh firmness and crispness retainability.

## Results

### Segregation and correlation of flesh firmness or crispness at harvest and post‐harvest

Both the initial flesh firmness and the crispness of apples at harvest segregated extensively with a Gaussian distribution within the three populations, implying a minor‐effect polygenic control of these two traits (Figure S1). The broad‐sense heritability (*H^2^*) was 75.24*–*83.06% and 86.91*–*94.43% for the initial flesh firmness and crispness, respectively (Table S1). Similarly, the *H^2^* was 75.04–83.85% and 75.87–83.64% for retainability of flesh firmness and crispness, respectively (Table S1). However, the frequency distributions of retainability of flesh firmness and crispness were not Gaussian (Figure S2). The majority of hybrids lost flesh firmness and crispness within one or two months. The frequency of hybrids with long flesh firmness (≥7 kg/cm^2^) and crispness (≥0.7 kg/cm^2^) retainability was higher in the population of ‘Zisai Pearl’ × ‘Red Fuji’ than the other two (Figure S2).

**Figure 1:**
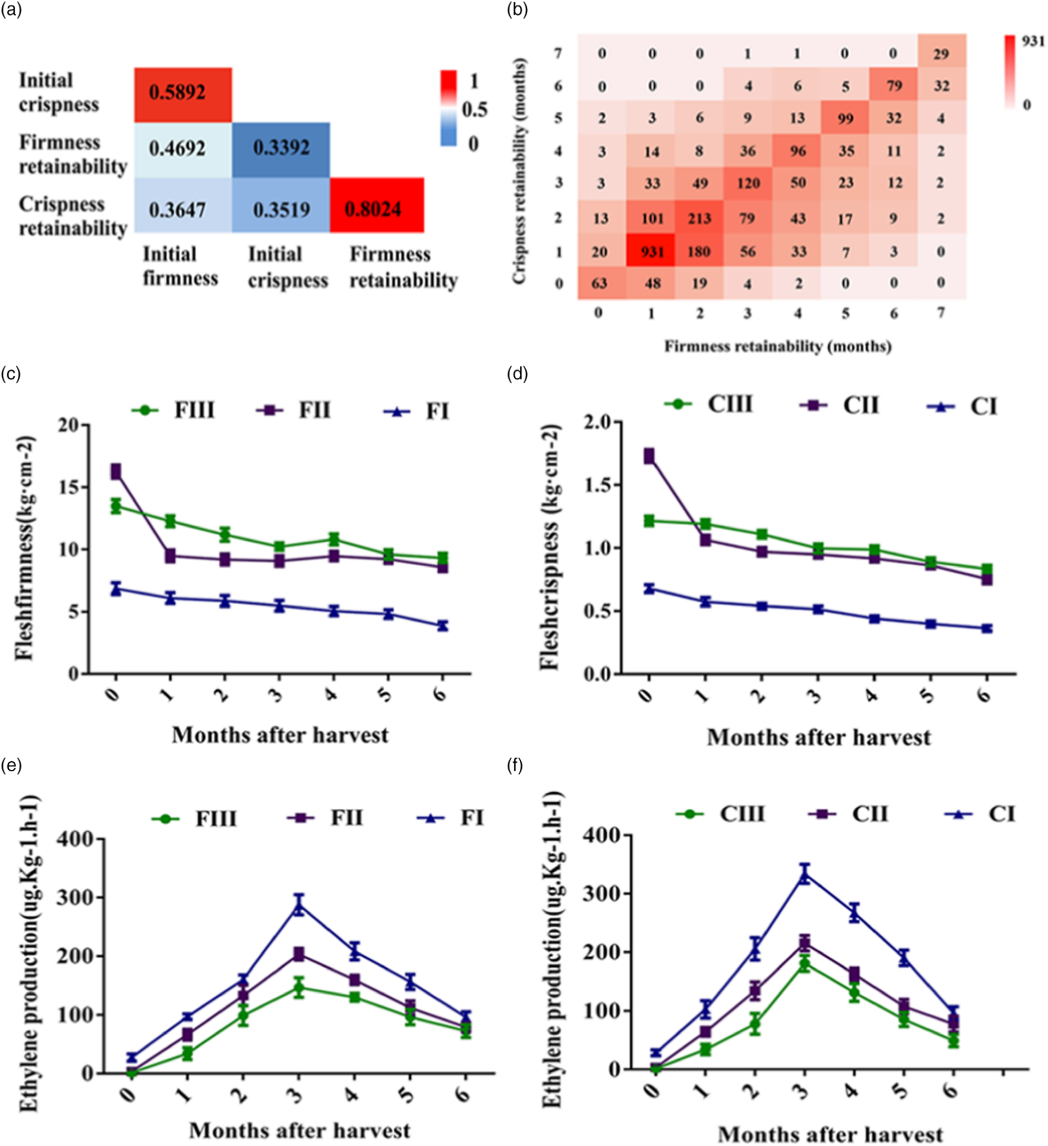
Changes and the relationship of apple flesh firmness and crispness retainability during cold storage in hybrid populations from ‘Zisai Pearl’ × ‘Red Fuji’, ‘Zisai Pearl’ × ‘Golden Delicious’ and ‘Jonathan’ × ‘Golden Delicious’. (a) Pearson’s correlation coefficients between phenotype properties of flesh firmness and crispness retainability. (b) Heatmap showing the consistency between flesh firmness retainability and crispness retainability. (c and d) The dynamic changes in flesh firmness (c) and crispness (d) during cold storage. (e and f) The dynamic changes in ethylene production during cold storage. FIII/CIII, FII/CII and FI/CI represent flesh firmness (F) and crispness (C) retainability phenotype type III, II and I, respectively.

The Pearson’s correlation coefficient was highest between flesh crispness and firmness retainability (0.8024; *n* = 2,651; *P* < 0.001), followed by that between initial flesh firmness and crispness (0.5892; *n* = 2,507; *P* < 0.001) (Figure 1a). The correlations between the initial flesh firmness or crispness at harvest and the corresponding flesh firmness or crispness retainability after cold storage were relatively low (0.3392–0.4692) (Figure 1a). The hybrids with long flesh firmness retainability did not generally have the same or longer flesh crispness retainability (Figure 1b). Among the 217 hybrids with 6–7 months long flesh firmness retainability, 140 (64.5%) had the same or longer flesh crispness retainability (Figure 1b). However, among the 157 hybrids with six months or longer flesh crispness retainability, 140 (89.2%) showed 6–7 months flesh firmness retainability (Figure 1b). These data indicated that long retainability of flesh firmness was likely a prerequisite for flesh crispness retainability.

### Pattern of flesh firmness and crispness at harvest and post‐harvest

Regarding the changes in flesh firmness and crispness during cold storage, three extremity dynamics were distinctly observed. Type I: acceptable flesh firmness (7.0 kg/cm^2^) or crispness (0.7 kg/cm^2^) lost already at harvest. Type II: acceptable flesh firmness or crispness maintained until the sixth month after a rapid softening during the first month of cold storage. Type III: acceptable flesh firmness or crispness maintained after the sixth months of cold storage with constantly slow softening rate (Figure 1c,d). Ethylene production in type I hybrids was higher than that in types II and III during cold storage, whereas type III hybrids produced lowest level of ethylene (Figure 1e,f). These suggested that low ethylene emission was associated with flesh firmness and crispness retainability.

### QTL identification using BSA‐seq

By the three segregant bulks from the three phenotype types of flesh firmness retainability (Figure S3a), 10, 5 and 17 significant QTLs for flesh firmness retainability were identified from bulk pairs of type III *vs*. type I, type III vs. type II, and type II vs. type I, respectively. These QTLs were located on chromosomes 3, 11, 12, 15, 16 and 17 (Figure S4a,b,c; Table S2). Of these QTLs, F‐F03.2 and F‐H03.1 overlapped at 28.5–29.8 Mb of chromosome 3, while F‐Z16.1, F‐F16.1, F‐H16.1 and F‐H16.2 overlapped at 38.4–39.5 Mb of chromosome 16 (Figure S5a,b; Table S2). From the three bulks of flesh crispness retainability (Figure S3b), 8, 11, and 11 QTLs were mapped from bulk pairs of type III *vs*. type I, type II *vs*. type I, and type III *vs*. type II, respectively. These QTLs were mapped on chromosomes 2, 3, 6, 10, 11, 14 and 16 (Figure S4d,e,f; Table S2). Of these QTLs, C‐H03.1, C‐F03.2 and C‐F03.3 overlapped at 28.4–29.7 Mb of chromosome 3. Similarly, C‐F16.1, C‐H16.1 and C‐H16.3 overlapped at 38.4–39.5 Mb of chromosome 16 (Figure S5a,b; Table S2). In addition, several QTLs for flesh firmness retainability on chromosome 3 and 16 overlapped with those for flesh crispness retainability, such as F‐F03.2/F‐H03.1/C‐H03.1/C‐F03.2/C‐F03.3 at 28.4–29.8 Mb of chromosome 3 (Figure S5a,b; Table S2). The overlap of these QTLs indicated the genetic linkage between flesh firmness and crispness retainability.

### Prediction of candidate genes for flesh firmness and crispness retainability at post‐harvest

By the RNA‐seq analysis, 47 222 unigenes were identified, of which 3750 differentially expressed genes (DEGs) were detected between the three types of flesh firmness and crispness retainability (Table S3). As expected, a clear transcriptomic signature of both the flesh firmness type I and the flesh crispness type I hybrids was the 2.18‐ to 56.19‐fold high expression of cell wall degradation‐related genes. These genes included *MdBGL* (MD00G1075400 and MD11G1023900), *MdBGAL* (MD15G1220900 and MD15G1221000) and *MdBGLU* (MD03G1215300) (Figure S6a,b; Tables S4 and S5). On the contrary, the expression of four cell wall‐associated genes was significantly higher (2.26‐ to 9.10‐fold difference) in flesh crispness retainable type III and type II hybrids than in crispness unretainable type I hybrids, that is *MdPGIP12* (MD05G1002900), *MdGLCAT14B* (MD02G1033100), *MdGWD1* (MD16G1097800) and a cellulolyase gene (MD17G1249600) (Figure S6b; Tables S4 and S5).

Several ethylene‐related DEGs were detected between flesh firmness type III or type II and type I hybrids. The expression of *MdZAT12* (MD01G1123300 and MD07G1192900), *MdERF110* (MD15G1334900) and *MdACO1* (MD15G1205100) was 0.01‐ *to* 0.48‐fold lower in type II and type III hybrids (Figure S6a; Tables S4 and S5). Similarly, the expression of *MdACO2* (MD05G1354000), *MdACO3* (MD02G1050800), *MdEIN3* (MD15G1441000) and *MdERF98* (MD06G1208700) was significantly low (0.01‐ to 0.47‐fold difference) in flesh crispness retainable type III and type II hybrids (Figure S6b; Tables S4 and S5). A group of ethylene response genes, however, was expressed more actively (2.02‐ *to* 2514.55‐fold difference) in firmness type III and type II hybrids than that in firmness type I hybrids, such as *MdAGL24* (MD15G1384500), *MdACO4* (MD16G1019900), *MdACO4‐like* (MD16G1019800), and *MdALD1* (MD15G1412200 and MD15G1412400).

High expression (2.95‐ to 37.14‐fold difference) of some auxin‐related genes, *MdSAUR71* (MD10G1202100), *MdSAUR40* (MD01G1106100) and *MdARGOS* (MD02G1000700), was observed in flesh firmness type I hybrids (Figure S6a; Table S4). In addition, a group of genes involved in abscisic acid (ABA) and gibberellic acid (GA) synthesis, transport, and response were differentially expressed between hybrid types of flesh firmness or crispness (Figures S6–S8; Tables S4 and S5). There were also DEGs detected between flesh firmness type III and type II hybrids, such as jasmonate (JA)‐related genes (MD07G1242100, MD07G1243000, MD07G1244000 and MD07G1244600) (Figure S6a; Tables S4 and S5). In summary, the above DEGs indicated the cell wall degradation, ethylene biosynthesis and signalling of ethylene, and other phytohormone were involved in flesh firmness and crispness retainability.

To predict candidate genes associated with flesh firmness and crispness retainability from QTL regions, 1865 and 1278 genes were downloaded from the intervals of the 62 QTLs for flesh firmness or crispness retainability, respectively. Then, 87 and 58 genes were selected according to the parental re‐sequencing and RNA‐seq data. These genes were functionally associated with fruit ripening and senescence (Table S6). From the QTL regions of F‐F03.1/C‐F03.2 and F‐Z16.2, two ERF genes, *MdERF3* (MD03G1194300) and *MdERF118* (MD16G1043500), were predicted as candidate genes for both flesh firmness and crispness retainability (Figure S5a,b; Table S6). *MdERF3* and *MdERF118* were also DEGs (Table S3).

### Validation of candidate genes *MdERF3* and *MdERF118*, and their interaction

**Figure 2:**
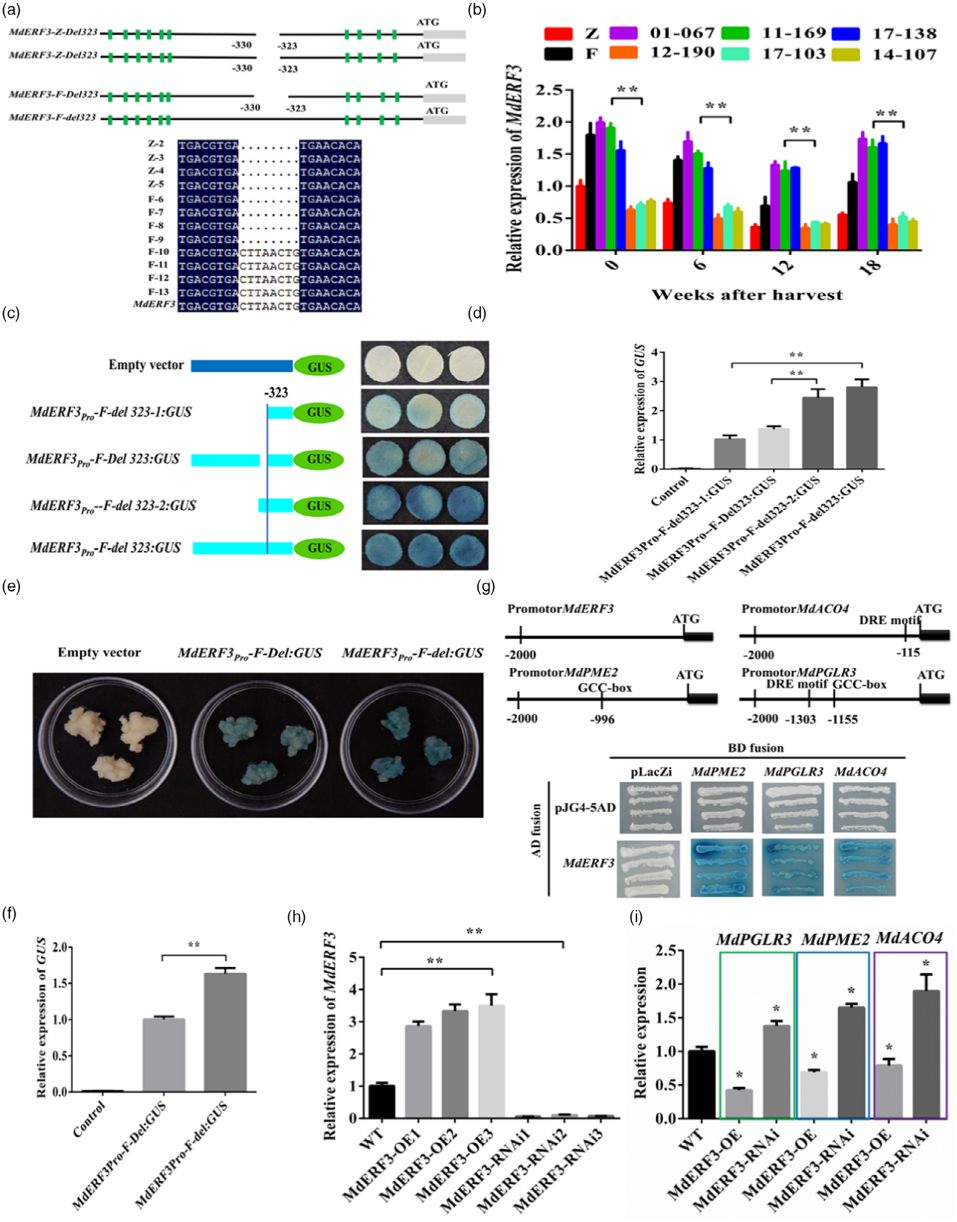
Functional validation of Del323 on the promoter of *MdERF3* in *Malus domestica* Borkh. ‘Red Fuji’ (F), *M. asiatica* Nakai ‘Zisai Pearl’ (Z), their F1 hybrids, and transgenic apple calli. (a) Diagram showing allelic variations in *MdERF3* promoter between ‘Z’ and ‘F’. The grey rectangle indicates coding sequence and the thick black line shows the upstream region. The gap on the line represents the 8 bp deletion at −323 to −330 bp (Del323) upstream of the ATG codon. The green rectangles indicate single‐nucleotide polymorphisms (SNPs). (b) *MdERF3* expression profiles in F1 hybrids with different flesh firmness/crispness retainability and their parents during cold storage. (c and d) Promoter truncation assay showing the promoter (2000 bp) activity *MdERF3* with or without Del323 using transgenic tobacco leaves by the β‐glucuronidase (GUS) staining (c) and GUS gene relative expression (d). (e and f ) GUS staining (e) and GUS gene expression (f) of *MdERF3* transformed apple calli with its own promoter. (g) Yeast one hybrid (Y1H) images showing the binding of MdERF3 protein to the promoters of *MdPGLR3, MdPME2,* and *MdACO4*. The upper part of this panel shows the locations of MdERF3 binding sites, GCC‐box and DRE motif, on the promoter of *MdPGLR3, MdPME2,* and *MdACO4*. (h and i) Relative expression of *MdERF3* (h), *MdPGLR3, MdPME2,* and *MdACO4* (i) in transgenic apple calli overexpressing *MdERF3* (*MdERF3*‐OE) or *MdERF3*‐RNAi lines. Wild type (WT) means untransformed control of apple callus. Statistically significant differences were determined by *t*‐tests: *P < 0.05, **P < 0.01.

An 8‐bp deletion (CTTAACTG) from −323 to −330 (Del323) prior to the ATG codon of *MdERF3* of ‘Red Fuji’ was detected and validated using Sanger sequencing (Figure 2a). The Del323 genotypes of ‘Red Fuji’ and ‘Zisai Pearl’ were heterozygous Del323:del323 and homologus Del323:Del323, respectively. Bioinformatic analysis predicted that this deletion might damage the DOF5.3 binding motif.

The expression of *MdERF3* in ‘Red Fuji’ and three flesh firmness and crispness retainable hybrids (01‐067, 11‐169, and 17‐138) with Del323:del323 genotype was significantly higher (2.1‐ to 2.9‐fold difference) than that in ‘Zisai Pearl’ and three flesh firmness and crispness non‐retainable hybrids (12‐190, 17‐103, and 14‐107) with Del323:Del323 genotype during 6 to 18 weeks of cold storage (Figure 2b).

β‐Glucuronidase (GUS) staining of transient transformed tobacco (*Nicotiana benthamiana*) leaves and the relative expression of *GUS* in *MdERFpro‐Fdel323:GUS* and *MdERF3_pro_‐Fdel323‐2:GUS* indicated that both the presence of Del323 from ‘Zisai Pearl’ and truncating off the corresponding fragment from ‘Red Fuji’ destroyed the promoter activity of *MdERF3* (Figure 2c,d). In transgenic apple calli, the relative promoter activity was significantly lower in *MdERF3_pro_‐F:GUS* (Del323) than in *MdERF3_pro_ ‐F:GUS* (del323) (Figure 2e,f).

Bioinformatics analysis predicted that *MdERF3* may bind to the GCC‐box or DRE motif on the promoter of genes such as *MdPGLR3, MdPME2* and *MdACO4* (Figure 2g)*. MdPGLR3, MdPME2* and *MdACO4* were predicted as candidate genes from QTLs F‐F03.4, C‐F02.1 and F‐Z16.1, respectively, and all these three genes were DEGs for flesh firmness and crispness retainability (Table S6). Yeast one hybrid (Y1H) assay confirmed that MdERF3 efficiently bound to the promoters of *MdPGLR3, MdPME2* and *MdACO4* (Figure 2g). The relative expression of *MdPGLR3, MdPME2* and *MdACO4* was significantly inhibited in *MdERF3* overexpressing lines, but was induced significantly in RNAi lines (Figure 2h,i). These data demonstrated that *MdERF3* might negatively regulate the transcription of *MdPGLR3, MdPME2* and *MdACO4*.

**Figure 3:**
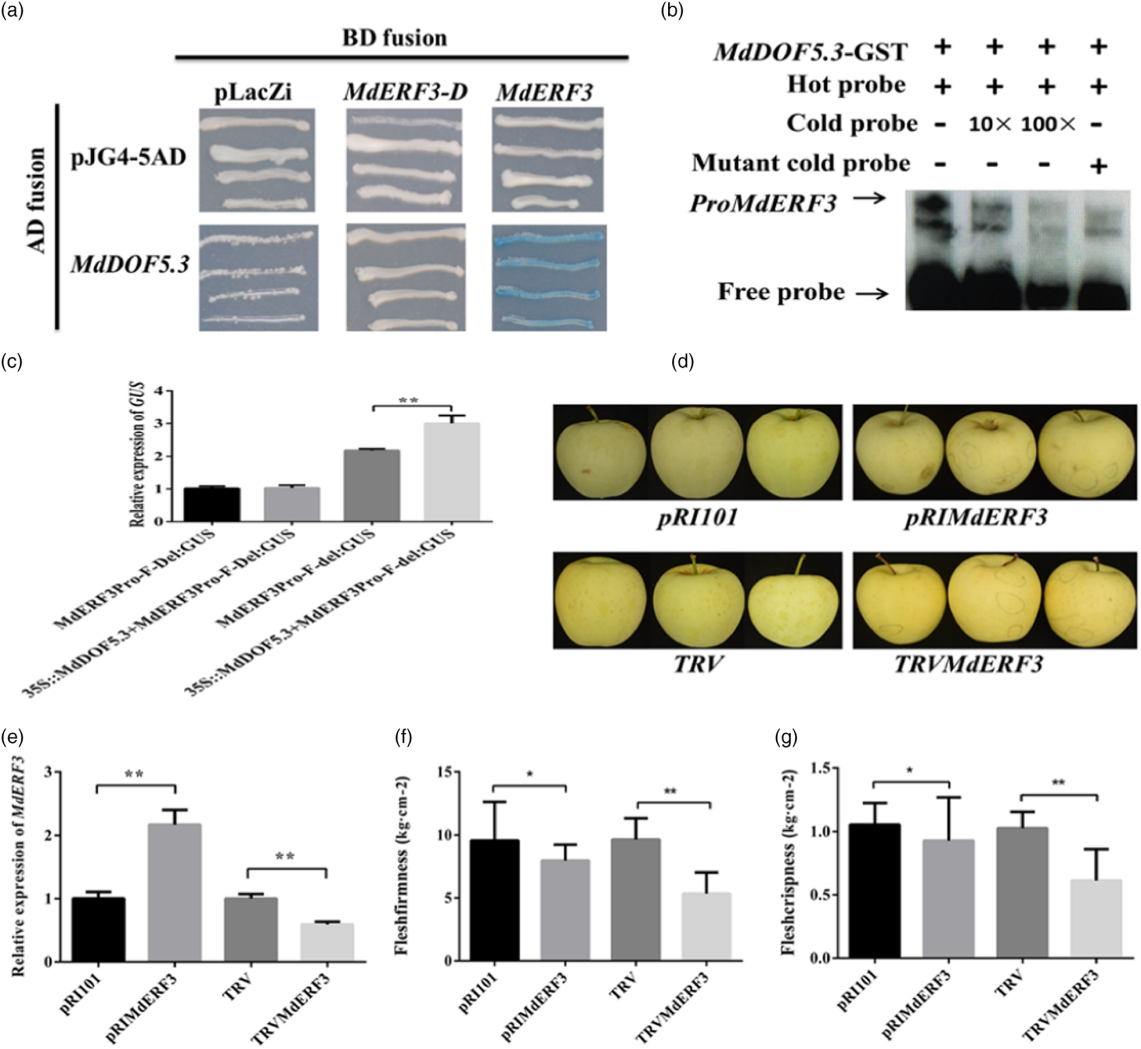
Validation of the interaction between MdDOF5.3 protein and *MdERF3* promoter and transiently overexpressing or virus‐induced gene silencing of *MdERF3* in ‘Golden Delicious’ apple. (a and b) Yeast one hybrid (a) and electrophoretic mobility shift assay (b) showing the interaction between MdDOF5.3 protein and the promoter of *MdERF3*. The hot probe in (b) was a biotin‐labelled *MdERF3* promoter fragment, and the cold probe was a unlabelled competitive probe (10 and 100‐fold that of the hot probe). A mutant cold probe (Mut) was an unlabelled hot probe sequence with two mutated nucleotides. GST‐tagged MdDOF5.3 was purified. (c) Relative expression of the β‐glucuronidase (GUS) gene in transiently co‐expressed 35S:MdDOF5.3 and *proMdERF3‐F:GUS* (del323) or *proMdERF3‐Z:GUS* (Del323) in tobacco (*Nicotiana benthamiana*) leaf. (d) Photographs showing apple skin colour changes after transient transformations. (e) The expression of *MdERF3* in apples overexpressing (*PRIMdERF3*) or silencing (*TRVMdERF3*) *MdERF3*. (f and g) Changes in flesh firmness (f) and crispness (g) in transiently transformed apples with empty vector (PRI101 and TRV), *PRIMdERF3* and *TRVMdERF3*. Asterisks indicate statistical significance (*P < 0.05, **P < 0.01).

*MdDOF5.3* was predicted to be located near QTL F‐Z11.1 (Table S6). Y1H revealed that MdDof5.3 bound to the promoter of *MdERF3* (del323) of ‘Red Fuji’ containing the CTTAACTG element, but did not bind to *MdERF3* (Del323) of ‘Red Fuji’ (Figure 3a). Electrophoretic mobility shift assay (EMSA) further confirmed the interaction between MdDOF5.3 and the promoter of *MdERF3* containing the CTTAACTG element (Figure 3b). In 35S:*MdDOF5.3* and *MdERF3_pro_‐F*:GUS (del323) co‐transformed tobacco leaves, the *MdERF3* promoter activity was higher than that co‐transformed with a *MdERF3_pro_‐F:*GUS (Del323) (Figure 3c). These results indicated that Del323 of *MdERF3* completely perturbed the binding of MdDOF5.3 to the CTTAACTG motif of *MdERF3* promoter.

*MdERF3* was then transiently overexpressed via *Agrobacterium*‐mediated infiltration or silenced via *pTRV* virus‐induced gene silencing (VIGS). Compared to that with the *PRI101* empty vector transformants control, a significant increase in *MdERF3* expression and distinct changes in fruit skin colour were observed in ‘Golden Delicious’ apples five days after transient transformation with *PRI‐MdERF3* (Figure 3d,e). However, both flesh firmness and crispness of *PRI‐MdERF*3‐transformed apples were relatively lower than that of *PRI101* control (Figure 3f,g), which may be attributed to natural fruit senescence. The apples transformed with *pTRV‐MdERF3* displayed more yellowing of the skin (Figure 3d), reduced *MdERF3* expression, and an especially lower flesh firmness and crispness five days after infiltration (Figure 3e–g). These data further confirmed that *MdERF3* contributed to post‐harvest flesh firmness/crispness retainability.

**Figure 4:**
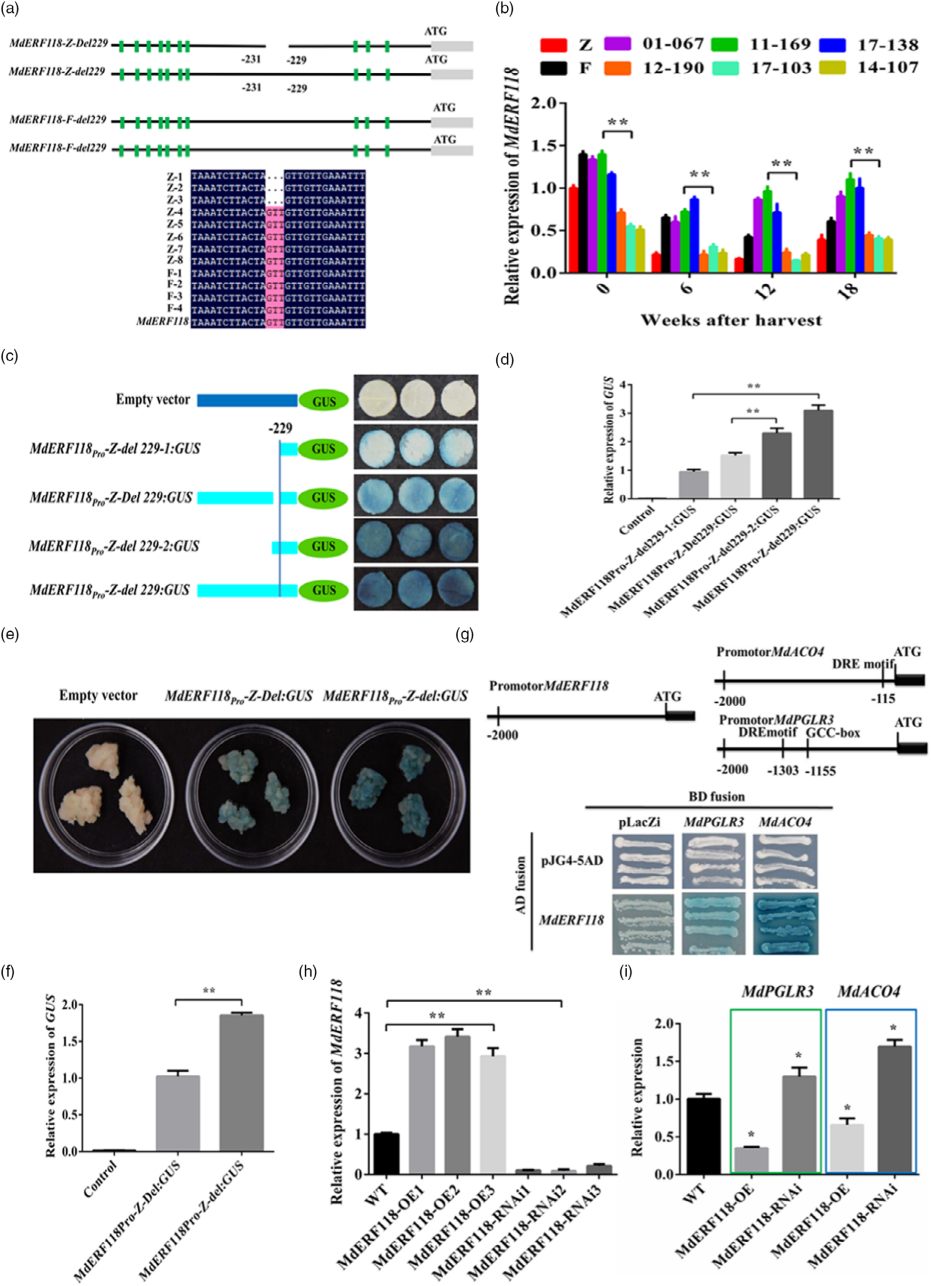
Functional validation of Del229 on the promoter of *MdERF118* in *Malus domestica* Borkh. ‘Red Fuji’ (F), *M. asiatica* Nakai ‘Zisai Pearl’ (Z), their F1 hybrids, and transgenic apple calli. (a) Allelic variations in *MdERF118* between ‘Z’ and ‘F’. The grey rectangle indicates coding sequence and the thick black line shows the upstream region, the gap represents the deletion (Del229) located at −229 to −231 bp upstream of the ATG codon, the green rectangles indicate single nucleotide polymorphisms (SNPs). (b) *MdERF118* expression in hybrids with different flesh firmness/crispness retainability and their parents during storage. (c and d) Promoter truncation assay showing the promoter (2000 bp) activity of *MdERF118‐Z* with (Del229) or without (del229) the Del229 using transgenic tobacco leaves by the β‐glucuronidase (GUS) staining (c) and GUS gene relative expression (d). (e and f) Images showing GUS staining (e) and GUS gene relative expression (f) of *MdERF118* transformed apple calli with its own promoter. (g) Yeast one hybrid analysis showing that MdERF118 protein binds to the *MdPGLR3* and *MdACO4* promoter containing the GCC‐box and DRE motif. The locations of GCC‐box and DRE motif on the promoter of *MdPGLR3* and *MdACO4* are shown at the upper part of the panel. (h and i) Relative expression of *MdERF118* (h), *MdPGLR3* and *MdACO4* (i) in transgenic apple calli overexpressing *MdERF118* (*MdERF118*‐OE) or *MdERF118*‐RNAi lines. Wild type (WT) means untransformed control of apple callus. Statistically significant differences were determined by *t*‐tests: *P < 0.05, **P < 0.01.

A 3‐bp deletion (GTT) at −229 to −231 (Del229) upstream of the ATG codon of *MdERF118* was detected in ‘Zisai Pearl’ (Figure 4a), which disrupted the RAVL1 binding CAACA motif and reduced the expression of *MdERF118* in flesh firmness and crispness non‐retainable Del229:del229 genotypes (Figure 4b). The GUS staining intensity, *GUS* gene expression in tobacco, and transgenic apple calli indicated that the presence of Del229 or truncating off the corresponding motif reduced the promoter activity of *MdERF118* (Figure 4c–f). Y1H and transgenic apple calli lines confirmed that *MdERF118* might negatively regulate the transcription of *MdPGLR3* and *MdACO4* (Figure 4g–i).

**Figure 5:**
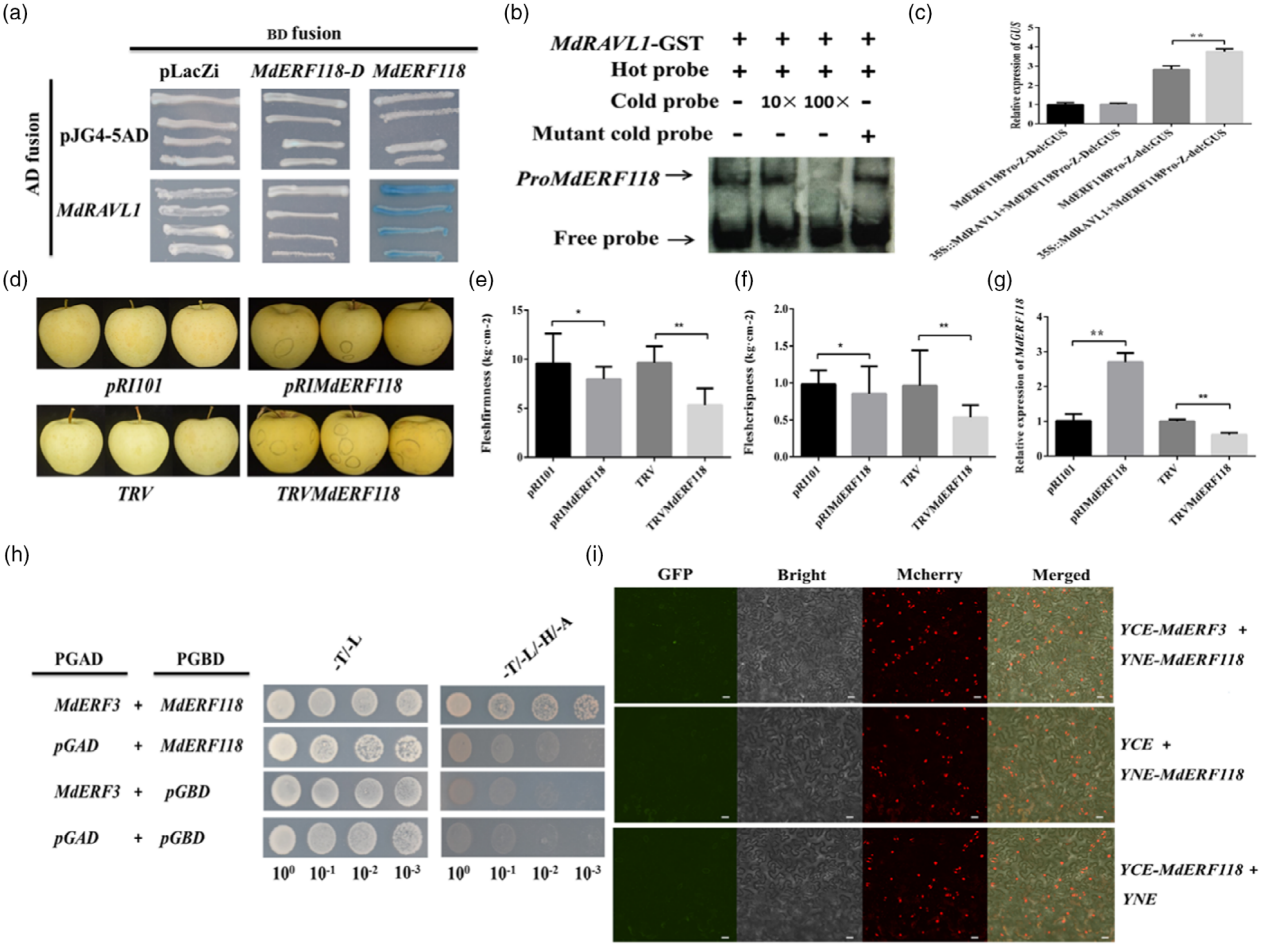
Validation of the interaction between MdRAVL1 protein and *MdERF118* promoter, and protein–protein interaction between MdERF3 and MdERF118. (a and b) Yeast one hybrid (a) and electrophoretic mobility shift assay (b) showing that MdRAVL1 binds only to the promoter of *MdERF118* promoter (del229). The hot probe in (b) was a biotin‐labelled *MdERF118* promoter fragment, and the cold probe was a unlabelled competitive probe (10 and 100‐fold that of the hot probe). A mutant cold probe (Mut) was an unlabelled hot probe sequence with two mutated nucleotides. GST‐tagged *MdRAVL1* was purified. (c) Relative expression of β‐glucuronidase (GUS) gene showing that MdRAVL1 induces the expression of *MdERF118‐F* (del229) but not *MdERF118‐Z* (Del229) in transiently co‐transformed tobacco (*Nicotiana benthamiana*) leaves. (d) Photographs showing apple skin colour changes after transient transformations of *MdERF118*. (e, f and g) Flesh firmness (e), crispness (f), and relative expression of *MdERF118* (g) in ‘Golden Delicious’ apple transiently transformed with empty vector (PRI101 and TRV), *PRIMdERF118* and *TRVMdERF118*. (h and i) Yeast two hybrid and bimolecular fluorescence complementation assay showing MdERF3 interacted with MdERF118. Gene fusion with vector pGBD or pGAD was used as negative controls in (h). The indicated split GFP constructs were transiently co‐expressed in *N. benthamiana* leaves in (i) and fluorescent images were obtained using confocal microscopy. Bars = 100 μm. Asterisks indicate significantly different values (*P < 0.05, **P < 0.01).

*MdRAVL1* was predicted from QTL F‐Z16.2 (Table S6). Y1H, EMSA and transient co‐transformed tobacco showed that MdRAVL1 bound to the promoter of *MdERF118* (del229) but not *MdERF118‐D* (Del229) (Figure 5a–c). ‘Golden Delicious’ apples transiently overexpressing or VIGS *MdERF118* further confirmed the contribution of *MdERF118* to flesh firmness/crispness retainability (Figure 5d–g).

The yeast two hybrid (Y2H) assay revealed the protein–protein interaction between MdERF3 and MdERF118 (Figure 5h). Then, a bimolecular fluorescence complementation (BiFC) assay in *N. benthamiana* leaves further verified that MdERF3 interacted with MdERF118 in the nucleus (Figure 5i).

### QTL‐based GAP modelling and simulative selection

**Figure 6:**
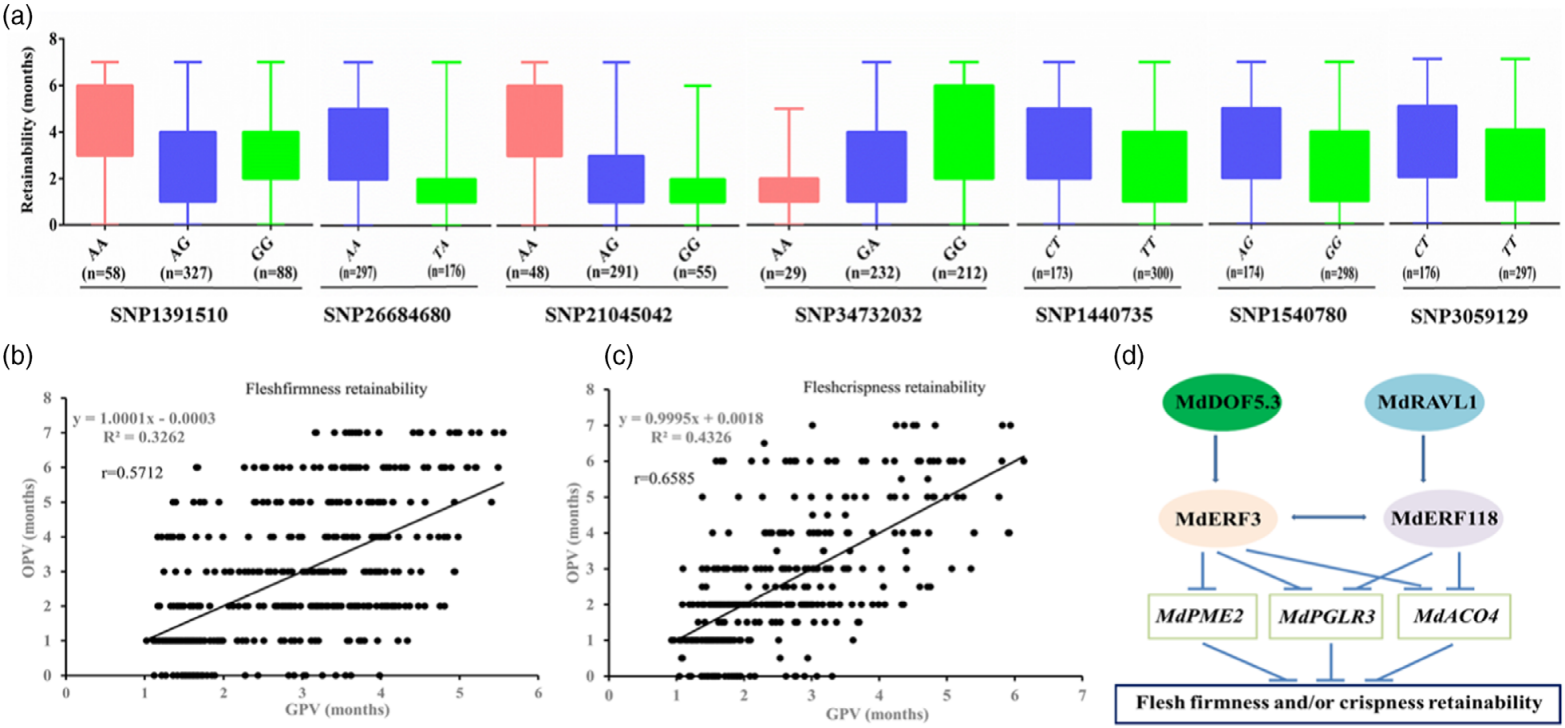
Marker genotype effect estimations (partially) (a), the linear regression between genomics prediction value (GPV) and observed phenotype value (OPV) of apple flesh firmness (b) and crispness (c) retainability, and the genetic variation network (d). The number in the parentheses under the genotypes in panel a means the number of hybrids with that genotype.

An SNP marker was selected from a candidate gene predicted from each of the 56 QTLs, including *MdERF3* and *MdERF118* (Figure S4; Table S7). The genotypes of hybrids in the training population for the 56 QTL‐based markers were listed in Tables S8 and S10. The marker effects varied from 0.155 months (SNP10070698) to 2.59 months (SNP9127269), and from 0.21 months (SNP10070698) to 3.24 months (SNP23366479) for flesh firmness and crispness retainability, respectively (Tables S9 and S11). The additive (e.g. SNP13579418 for crispness retainability) or complete dominant (e.g. SNP9403318 for crispness retainability) allelic effects were less common than the partial dominant allelic effect (e.g. SNP9127269 and SNP38573461 for both firmness and crispness retainability) (Figure 6a).

The genomics‐predicted value (GPV) of flesh firmness retainability of 473 hybrids and GPV of flesh crispness retainability of 394 hybrids were shown in Tables S8 and S10, respectively. Relatively high prediction accuracy was obtained as 0.5712 and 0.6585 for the retainability of flesh firmness and crispness, respectively (Figure 6b,c). The fivefold cross‐validation exhibited an average prediction accuracy of 0.5541 and 0.6018 for flesh firmness and crispness retainability, respectively (Tables S12 and S13). In a simulative selection, when flesh firmness retainability GPV criterion was set as 5.0 months, the selection rate was 2.54% and the efficiency was 100% (12/12), which indicated that the observed phenotype value (OPV) of all the 12 selected hybrids was equal to or longer than 5.0 months (Table S14). When the GPV criterion for flesh crispness retainability was set as 5.0 months, the selection rate was 3.48% and the efficiency was 64.29% (Table S15).

## Discussion

Flesh firmness retainability and crispness retainability are the critical properties affecting fruit cold storability. The heritability of flesh firmness and crispness retainability was as high as 83.85% and 83.64%, respectively, which was slightly higher than the estimate (70.0%) for firmness and crispness at harvest or after two month cold storage (Bink *et al*., 2014; McKay *et al*., 2011). Flesh firmness and crispness retention can be prolonged if the initial values of firmness and crispness at harvest are high or if the flesh softening rates during storage are low, or both (Costa, 2015; Johnston *et al*., 2001; Nybom *et al*., 2013). In this study, unlike flesh firmness and crispness at harvest, the segregation of flesh firmness and crispness retainability did not displayed Gaussian distribution, a large proportion of hybrids lost acceptable flesh firmness and crispness one month post‐harvest.

According to the QTL effects in tomato, flesh firmness loss was not predicted by firmness at harvest (Aurand *et al*., 2012). The phenotype correlations between the initial flesh firmness/crispness and their retainability were also relatively low in this study. In a two‐year trial, apple fruit firmness after storage correlated positively with both firmness at harvest and changes in firmness during storage; however, no obvious correlation was observed between changes in firmness and the initial firmness at harvest (McClure *et al*., 2018). A major QTL for apple fruit firmness retention after storage was identified on chromosome 10; however, a gene encoding ERF (MDP0000855671) was predicted as the candidate rather than the frequently detected *MdPG1* in other populations, indicating that the genetics of the trait firmness retention might differ from that of initial firmness at harvest (McClure *et al*., 2018). In a MetaQTL analysis, of the six MetaQTLs for apple fruit texture attributes, two (MetaQTL6.1 and MetaQTL10.1) were for both firmness at harvest and firmness after two months cold storage, one (MetaQTL10.2) for both softening and firmness after two months, and three for solely softening (MetaQTL5.1), firmness at harvest (MetaQTL15.3), and firmness after two months (MetaQTL15.1), respectively (Costa, 2015). This indicated that these three texture attributes were mutually related but partially independent. High initial flesh firmness or crispness is necessary but insufficient for long‐term firmness and crispness retainability. In this study, several new QTLs for flesh firmness and crispness retainability were identified on chromosomes 2, 11, 12 and 17, which were previously considered as always having low values for segregating QTLs on these chromosomes (Bink *et al*., 2014).

The four functional markers, *MdACS1, MdACO1, MdPG1* and an expansin gene, *MdExp7*, for fruit firmness and crispness in apple have been well studied to dissect phenotypic variability among cultivars and hybrids (Costa *et al*., 2005a, 2008; Harada *et al*., 2000; Wang *et al*., 2009). Unfortunately, the empirical predictive power of these four functional markers were limited, with 15% of the observed variation in initial firmness and 18% in softening rate; therefore, their large‐scale use for MAS was doubtful (Nybom *et al*., 2013). The genotypes of commonly used markers on *MdACS1* (MD15G1302200) and *MdACO1* (MD15G1205100) did not segregate in the ‘Zisai Pearl’ × ‘Red Fuji’ population (Wu et al., 2020), which offered opportunity to map more QTLs. Ethylene production, however, varied with types of firmness and crispness retainability (Figure 1e,f). RNA‐seq data showed that the expression levels of *MdACO1*, *MdACO2*, *MdACO3* and some ethylene response genes were higher in flesh firmness and crispness unretainable type I hybrids.

Flesh firmness and crispness are usually measured simultaneously; however, they should be two closely relevant but clearly distinct texture attributes (Nybom *et al*., 2013). One evidence for the close relatedness was that the phenotypic correlation coefficient between the flesh firmness retainability and crispness retainability was as high as 0.8024, which was inconsistent with the previous report (Hardner *et al*., 2016). Another evidence was that the overlapping QTLs on chromosomes 3 and 16 associated with flesh firmness and crispness retainability. The third evidence was that variations in *MdERF3* and *MdERF118* affected both flesh firmness and crispness. On the other hand, retainability of flesh firmness was likely a prerequisite for flesh crispness retainability. Additionally, many QTLs were identified independently for flesh firmness retainability and crispness retainability, which manifested the distinctness of these two flesh texture attributes.

The majority of consumers prefer crisp apples to firm ones (Bonany *et al*., 2014; Hoehn *et al*., 2008; Hong *et al*., 2018; Yue *et al*., 2013). Apple cultivars with relatively high flesh crispness but low firmness, such as 'Honeycrisp', are currently commercially successful and are expected to remain so in the future (McKay *et al*., 2011). The apple expansin genes, *MdEXPDCA1*, *MdEXPA2*, *MdEXPA3* and *MdEXPA8* were considered markers for flesh crispness retention during cold storage (Costa *et al*., 2005b; Trujillo *et al*., 2012). We observed that the expression of β‐Gal genes, *MdBGLs* and *MdBGLU*, was significantly high in flesh firmness and crispness unretainable hybrids, which was consistent with the results of a previous report (Wei *et al*., 2012). These data indicated that flesh crispness retainability is a more crucial fruit quality attribute than flesh firmness retainability and that genomics‐assisted selection for good flesh crispness retainability may also inadvertently select for good flesh firmness retainability.

Unlike the QTLs on chromosomes 10 and 15 for fruit firmness at harvest, chromosomes 3 and 16 were ‘QTL hotspots’ for apple flesh firmness and crispness retainability (Figure S5a,b), which is consistent with the results of previous reports (Bink *et al*., 2014; Di Guardo *et al*., 2017). The markers SNP26684680 and SNP34732032 on chromosome 3, and SNP38573461, SNP3059129, and SNP9127269 on chromosome 16 accounted for relatively large effects for both flesh firmness and crispness retainability (Figure S4; Tables S9 and S11).

RNA‐seq enriched a large group of ethylene synthesis‐related and cell wall degradation‐related genes in DEGs between flesh firmness and crispness retainable and unretainable samples, which was consistent with previous reports (Dandekar *et al*., 2004; Gwanpua *et al*., 2014; Longhi *et al*., 2013; Wakasa *et al*., 2006). In addition to these functional genes, many ethylene receptors and signalling genes were involved in regulating fruit firmness, such as *MdEIN3*, *MdERF1*, *MdERF2* and *MdERF4* (Lee *et al*., 2012; Li *et al*., 2017). Here, we observed that several ethylene‐responsive genes were up‐regulated in firmness and crispness retainable apples (Figure S6). As expected, the allelic variations on QTL‐identified *MdERF3* and *MdERF118* were detected and were validated to affect flesh firmness and crispness retainability (Figure 6a). These two allelic variations were considered diagnostic markers, which is consistent with QTL‐derived *ERF* genes for apple fruit firmness retention (McClure *et al*., 2018). MdERF3 contains an ethylene‐responsive element‐binding factor‐associated amphiphilic repression (EAR) motif, and thus may function as negative regulator of *MdPGLR3, MdPME2* and *MdACO4* expression (Kagale and Rozwadowsld, 2011). *MdERF118* also inhibited *MdPGLR3* and *MdACO4* expression by directly binding to their promoters; however, *MdERF118* does not contain an EAR motif, which is similar to *SlERF6* in tomato. *SlERF6* negatively regulates fruit maturity and does not contain the EAR motif (Lee *et al*., 2012). The protein–protein interaction between MdERF3 and MdERF118 rendered the genetic variation network more complex (Figure 6d).

GAP models for apple flesh firmness and crispness retainability were developed and were efficiently used in simulative selection. Fivefold cross‐validation showed the prediction accuracy was 0.5541 and 0.6018 for the retainability of flesh firmness and crispness, respectively. The predictability of these models was not only better than that of the activator‐depleted substrate model (van der Sman and Sanders, 2012), but also better than that of pure GS by ridge regression BLUP, the accuracy of which was more than 0.4 for apple firmness after storage and was 0.08–0.45 for changes in fruit firmness (McClure *et al*., 2018). The improvement in predictability of the GAP models may be attributed to the taking into account non‐additive allelic effects. Additive, dominant, and even over‐dominant non‐allelic epistasis or interactions among QTLs or candidate genes for fruit firmness were detected in tomato (Causse *et al*., 2007; Chapman *et al*., 2012). Dominant or partially dominant allelic effects were common in this study for apple flesh firmness and crispness retainability.

## Conclusion

Fifty‐six candidate genes for apple flesh firmness and crispness retainability were predicted from the intervals of 62 QTLs. GAP models were developed using genotype effects of the QTL‐based marker, and the prediction accuracy of the GAP models was 0.5712 and 0.6585 for the retainability of flesh firmness and crispness, respectively. On the QTL hotspot of chromosome 3, an 8‐bp deletion in the *MdERF3* promoter perturbed the binding of MdDOF5.3, reduced *MdERF3* expression, relieved the inhibition on *MdPGLR3*, *MdPME2*, and *MdACO4* expression, and ultimately decreased the flesh firmness and crispness retainability. From a QTL on chromosome 16, a 3‐bp deletion in the promoter of *MdERF118* decreased its expression by perturbing the binding of MdRAVL1, which increased *MdPGLR3* and *MdACO4* expression and reduced flesh firmness and crispness retainability. The results implied a sophisticated genetic variation network regulating flesh firmness and crispness retainability. The GAP models can be potentially applied in apple breeding.

## Experimental procedures

### Plant materials and growth conditions

The plant materials were 2,664 bi‐parental hybrids derived from ‘Zisai Pearl’ × ‘Red Fuji’ (1185), ‘Zisai Pearl’ × ‘Golden Delicious’ (967), and ‘Jonathan’ × ‘Golden Delicious’ (512). ‘Zisai Pearl’ is a Chinese apple cultivar that belongs to *Malus asiatica* Nakai., while the other three parents are commercial cultivars belonging to *M. domestica* Borkh. All the hybrids were grown at a density of 2.5 m × 0.5 m under conventional management and pest control in the Fruit Experimental Station, China Agricultural University (Changping District, Beijing, China).

In 2016–2017, fruit samples were harvested at commercial maturity based on fruit background colour and starch degradation (selected at 7 on a scale of 1–10) (Blanpied and Silsby 1992). After harvest, apples were cold‐stored at 1 °C with −95% relative humidity in polyethylene bags. Flesh firmness and crispness of three apples per hybrid (all 2,664 hybrids if fruit samples were available) were measured at 0–6th month with one month interval. Apples from hybrids with year‐stable extremity phenotypes (randomly five hybrids from each phenotype type) were used for ethylene measurement in 2017. Flesh tissue of ‘Zisai Pearl’, ‘Red Fuji’ and hybrids with phenotype type I (12‐190, 17‐103, and 14‐107) and type II (01‐067, 11‐169, and 17‐138) of flesh firmness retainability were used for qRT‐PCR analysis for *MdERF3* and *MdERF118* relative expression. The fruit flesh and leaf samples were collected, immediately frozen in liquid nitrogen and stored at −80 °C until use.

Apple calli induced from the flesh of the cultivar ‘Orin’ were sub‐cultured every three weeks on Murashige and Skoog medium used for *A. tumefaciens* infection (Jia *et al*., 2018).

### Measurements of flesh firmness/crispness and the retainability

To evaluate the ability of flesh firmness and crispness retention during cold storage, the maximum time (months as unit) till which the apples maintained acceptable flesh firmness (≥7.0 kg/cm^2^) and crispness (≥0.7 kg/cm^2^) was recorded as the retainability of flesh firmness and crispness, respectively (Costa *et al*., 2010; Nybom *et al*., 2012). Flesh firmness and crispness were measured with a computer‐controlled texture analyser TAXT (StableMicroSystem, Godalming, UK) (Costa *et al*., 2011, 2012). The diameter of the penetrating probe was 0.2 cm, and the penetration depth was 0.5 cm. Punctures were performed on the maximum equator of each apple. Three biological replicates were performed, and each included at least three technical replicates.

### Measurements of ethylene production

To determine ethylene emission, each fruit was weighed and enclosed in a gas‐tight container and incubated for 2 h at room temperature (20–25 °C). One millilitre of gas was sampled from the headspace in the container using a BD syringe (No. 309602, BD, Franklin Lakes, NJ, USA). The ethylene concentration of gas samples was measured with a gas chromatography (HP 5890 series II) (Hewlett‐Packard, Palo Alto, CA, USA) equipped with a flame ionization detector. The fruit ethylene production was calculated as described previously (Dougherty *et al*., 2016).

### BSA‐seq and QTL identification

As hybrids with longer flesh firmness and crispness retainability over six months can only be detected in the population of ‘Zisai Pearl’ × ‘Red Fuji’, hybrids from this population were used to make BSA bulks. Thirty hybrids were randomly chosen from each phenotype type: type I, type II, and type III for flesh firmness or crispness retainability, respectively. Totally six phenotype extremity bulks were constructed (Figure S3). Genomic DNA from each hybrid was extracted from young leaves and pooled. Each bulked DNA sample was sequenced to obtain 30× genome coverage using a paired‐end 150‐bp read strategy (Illumina X10, Illumin a, USA). The parental cultivars have been re‐sequenced previously (Shen *et al*., 2019; Wu et al., 2020). The re‐sequencing data were processed, and QTLs were identified using the BSATOS software package (https://github.com/maypoleflyn/BSATOS) (Shen *et al*., 2019).

### RNA‐seq

RNA‐seq was performed to explore DEGs between phenotype types of flesh firmness and crispness retainability during cold storage. Apples of three hybrids (as three biological replicates) from each phenotype type, III, II, and I of flesh firmness or crispness retainability were respectively sampled at 0, 6, 12 and 18 weeks of cold storage. Totally, 72 samples were collected. The Illumina HiSeq 2000 platform was used to generate 100‐bp paired‐end reads. Bioinformatic analysis of RNA‐seq data was performed as previously described (Sun *et al*., 2016). DEGs were determined by using the false discovery rate (FDR) <0.05 and the fold change threshold >2.0.

### Candidate gene prediction

To develop potential diagnostic markers, candidate genes associated with flesh firmness and crispness retainability were predicted by using multi‐omics data (Shen *et al*., 2019). Genes were downloaded from the intervals of the QTLs for flesh firmness or crispness retainability, respectively. SNPs and insertion/deletion variations (InDels) in these genes were called using parental re‐sequencing data. Of these genes, those with SNPs or InDels that did not affect the cis‐element on the upstream sequence or the functional domain on the coding region were excluded. The genes which expression was not detectable, in addition to the genes with SNPs or InDels only in the promoter but that were not included in the DEGs, were excluded from further analysis.

### Genomic DNA extraction, RNA extraction and qRT‐PCR assay

Genomic DNA of young leaves and total RNA of apple flesh or apple calli samples were extracted using the modified CTAB method as previously described (Gasic *et al*., 2004; Muhammad *et al*., 1994). cDNA was synthesized using a cDNA synthesis kit (Takara, Japan). The primers were designed according to the sequence on apple genome (GDDH13 v1.1) with premier software (version 5.0) (Premier Biosoft Interpairs, Palo Alto, CA) (Daccord *et al*., 2017). The qRT‐PCR analysis was performed using the ABI PRISM 7500 real‐time PCR system (Applied Biosystems). The gene encoding apple actin was used as the internal control. Three biological and three technical replicates (3 × 3) were performed. The primer sequences are listed in Table S16.

### Functional validation of candidate genes

#### GUS analysis

To test whether the allelic variations upstream of *MdERF3* and *MdERF118* coding sequences (CDS) may affect its promoter activity, 2000‐bp promoter fragments were amplified using PCR from the genomic DNA of ‘Zisai Pearl’ and ‘Red Fuji’. After Sanger sequencing, the reporter constructs containing the promoter sequences of *MdERF3* and *MdERF118* (2000 bp upstream of the start ATG codon, using restriction enzyme sites SalI and EcoRI) were prepared as previously described (Jia *et al*., 2018). The CDS of *MdDof5.3* or *MdRAVL1* was introduced into the pRI101 vector using restriction enzyme sites (SalI and BamHI for *MdDof5.3*; BamHI and XholI for *MdRAVL1*) to generate the effector constructs. The reporter and effector vectors were constructed and injected into tobacco leaves. After three days infection, the transgenic leaves were dyed using the GUS reporter gene staining kit (Solarbio, China) in three biological replicates and four technical replicates, as described by Zhang *et al*. (2016). All primer sequences for vector construction are listed in Table S16.

#### Transient overexpression and VIGS

A 690 bp *MdERF3* and a 1032 bp *MdERF118* CDS fragments were cloned from ‘Golden Delicious’ into the EcoRI/XholI and EcoRI/KpnI sites of the pTRV2 virus vector as previously described (Li *et al*., 2017). *A. tumefaciens* cells harbouring the resultant plasmids were suspended in infiltration buffer supplemented with 150 mm acetosyringone. The inoculum preparations were adjusted to OD_600_ = 1.0. A mixture of *A. tumefaciens* cells harbouring pTRV1 and pTRV2 derivatives (1: 1 ratio) was infiltrated into ‘Golden Delicious’ apple fruit (140 days after anthesis) (Li *et al*., 2016). The complete *MdERF3* and *MdERF118* CDS cDNA sequences were amplified and individually cloned into the NdeI/EcoRI and SalI/BamHI sites of the PRI101 vector, followed by infiltration into ‘Golden Delicious’ apple fruit. The assays were performed with at least six fruit for each vector, and the experiments were repeated at least three times. The primer pairs used are listed in Table S16.

#### Apple calli transformation

To determine the impact of the allelic variations in the upstream regions of *MdERF3* and *MdERF118* on promoter activity, genetic constructs with the promoters of *MdERF3* and *MdERF118*, and also their CDS, respectively, plus a GUS reporter, were prepared using the one‐step seamless cloning kit (Aidlab Biotechnologies, Beijing, China). These included *MdERF3_pro_‐F:GUS* (Del323), *MdERF3_pro_‐F:GUS* (del323), *MdERF118_pro_‐Z:GUS* (Del229), and *MdERF118_pro_‐Z:GUS* (del229). All constructs were transiently transformed into apple calli (from hypanthium of ‘Orin’ cultivar) using the method described by Jia *et al*. (2018). The complete *MdERF3* and *MdERF118* CDS cDNA sequences were also amplified and cloned into the NdeI/EcoRI and SalI/BamHI sites of the PRI101 vector, respectively. Partial *MdERF3* and *MdERF118* CDS sequences were cloned using the restriction enzyme sites XbaI/SalI, EcoRI/SalI, and XbaI/SalI, KpnI/EcoRI of the RNAi vector. The primer pairs used are listed in Table S16.

#### Y1H assay

The CDS of *MdERF3* and *MdERF118* were ligated into the PJG4‐5 vector in restriction enzyme sites EcoRI/XholI. *MdPME2, MdPGLR3* and *MdACO4* were ligated into the Placzi vector in restriction enzyme sites EcoRI/XholI, KpnI/XholI, and KpnI/XholI, respectively. The CDS of *MdDof5.3* and *MdRAVL1* were ligated into the PJG4‐5 vector using restriction enzyme sites EcoRI/XholI. *MdERF3* and *MdERF118* were ligated into the Placzi vector, using restriction enzyme sites EcoRI/XholI. All primers used are listed in Table S16.

#### EMSA

The proteins of MdDof5.3 and MdRAVL1 were induced at 37 °C for 6 h. Proteins were purified as described by Li (Li *et al*., 2016). Oligonucleotide probes were synthesized and labelled with biotin (Sangon Biotech.Co., Ltd., Shanghai, China). The biotin end‐labelled double‐stranded DNA probes were prepared by annealing complementary oligonucleotides as follows: the oligonucleotides were heated at 95 °C for 5 min, then at 72 °C for 20 min, and immediately left to cool to room temperature before use. The biotin‐labelled *MdERF3* promoter and *MdERF118* primer sequences are shown in Table S16.

#### Y2H assay

The CDS region of *MdERF3* was ligated into the pGADT7 vector using the EcoRI and XholI restriction sites as described previously (Jia *et al*., 2018). The full‐length *MdERF118* (1−344 amino acids) was ligated into the pGBKT7 (Clontech, Beijing, China) binding domain (BD) vector using the EcoRI and SalI sites. The primers used are shown in Table S16. BD and AD vectors were co‐transformed into the Y2HGold yeast strain. The interactions between two proteins were detected using the Matchmaker Gold Y2H library screening system (catalog no. 630489; Clontech, Beijing, China).

#### BiFC assay

The full‐length CDS of *MdERF3* or *MdERF118* without stop codon was amplified and ligated into pSPYNE‐35S and pSPYCE‐35S vectors containing green fluorescent protein (GFP), using the one‐step seamless cloning kit (Aidlab Biotechnologies); the constructs were checked by sequencing and then transformed into *A. tumefaciens* strain GV3101. Two plasmids were co‐transformed into the abaxial side of 4‐ to 6‐week‐old *N. benthamiana* leaves to detect specific interactions as described previously (Zhang *et al*., 2013). After 48 h co‐infiltration, the *N. benthamiana* leaves were observed using an LSM 510‐Meta confocal laser microscope (Zeiss). GFP signals were imaged under 488 nm excitation wavelength.

### Kompetitive allele‐specific PCR (KASP) genotyping and GAP modelling

Three hundred hybrids were randomly selected from each F1 population of ‘Zisai Pearl’ × ‘Red Fuji’, ‘Zisai Pearl’ × ‘Golden Delicious’, and ‘Jonathan’ × ‘Golden Delicious’. All these hybrids were genotyped for the 56 markers (29 for flesh firmness retainability and 27 for flesh crispness retainability) using KASP assay. The 200‐bp sequences flanking the interested SNPs were used for KASP primer design (Chen *et al*., 2011). The KASP primers are listed in Table S7. The KASP assay was performed using the LGC SNPline (Laboratory of the Government Chemist, UK) and the data were analysed using SNPviewer software. The DNA of parental cultivars, ‘Jonathan’, ‘Golden Delicious’, ‘Zisai Pearl’, and ‘Red Fuji’, were used as control in KASP assay, because the marker genotypes of these cultivars were already known by previous parental re‐sequencing (Shen *et al*., 2019).

Marker genotype effect was estimated by the deviation between the average OPV of the hybrids with the same genotype and overall mean phenotype value of the training populations. By using hybrids without missing marker genotype data, the GPV was calculated by sum up the genotype effects of all markers and population mean phenotype value. The prediction accuracy of GPV was evaluated by the Pearson’s correlation coefficient between GPV and OPV of the hybrids (Kumar *et al*., 2012a). Fivefold cross‐validation (run five times) was used to test the accuracy of the GAP models (Abed *et al*., 2018; Jiang *et al*., 2017; Kumar *et al*., 2012a).

### Statistical analysis

Significant differences were analysed using the independent samples Student’s *t* test (SPSS Statistics 21; IBM). Asterisks denoted significance: *P < 0.05, **P < 0.01. Error bars indicate standard deviation of three biological replicates, each containing at least three technical replicates.

## Accession numbers

Sequence data were based on online databases (https://www.uniprot.org/ and https://www.rosaceae.org/species/malus/malus × domestica, GDR). All BSA‐seq and RNA‐seq raw data have been deposited in the NCBI Sequence Read Archive (SRA) under the accession number PRJNA650592. The accession numbers for the genes in this study are as follow: *MdPME2* (MD02G1207900), *MdPGLR3* (MD03G1260700), *MdACO4* (MD16G1019900), *MdDOF5.3* (MD11G1123400), *MdRAVL1* (MD16G1047700), *MdERF3* (MD03G1194300) and *MdERF118* (MD16G1043500).

## Conflicts of interest

The authors declare that they have no competing interests.

## Author contributions

B.W. analysed all the data and wrote the draft manuscript. X.Z.Z. and Z.H.H. designed and supervised the experiments. X.Z.Z. revised the manuscript. F.S. gave advice for bioinformatic analysis. X.W., W.Y.Z., C.X., Y.D., T.W., Z.Y.H. and Q.Z. collected the apple samples. Y.W., T.W. and X.F.X. prepared the plant materials. All authors read and approved the manuscript.

## Supporting information


**Figure S1** Frequency distribution of initial flesh firmness and crispness at harvest of hybrids from ‘Zisai Pearl’ (Z) × ‘Red Fuji’ (F), ‘Zisai Pearl’ × ‘Golden Delicious’ (G) and ‘Jonathan’ (J) × ‘Golden Delicious’ in 2016‐1017.
**Figure S2** Frequency distribution of apple flesh firmness and crispness retainability of hybrids from ‘Zisai Pearl’ (Z) × ‘Red Fuji’ (F), ‘Zisai Pearl’ × ‘Golden Delicious’ (G) and ‘Jonathan’ (J) × ‘Golden Delicious’ in 2016‐1017.
**Figure S3** Flesh firmness and crispness of the segregant bulks at the sixths month of cold storage from hybrids of ‘Zisai Pearl’ × ‘Red Fuji’. (a) The bulks for type III, type II and type I of flesh firmness retainability. (b) The bulks for type III, type II and type I of flesh crispness retainability.
**Figure S4** Quantitative trait loci (QTLs) for apple flesh firmness and crispness retainability identified via BSA‐seq using hybrids from *Malus asiatica* Nakai. ‘Zisai Pearl’ × *M. domestica* Borkh. ‘Red Fuji’. (a, b and c) QTLs for flesh firmness retainability by pairwise comparisons between phenotype type III (FFIII) and type I (FFI) (a), type III and type II (FFII) (b), or type II and type I (c). (d, e and f) QTLs for flesh crispness retainability comparison between phenotype type III (FC‐III) and type I (FC‐I) (d), type II (FC‐II) and type I (e), or between type III and type II (f). The solid horizontal lines represent significant thresholds for QTLs on the maternal (red), paternal (blue) and both (black) parents.
**Figure S5** Overlapping of QTLs on chromosome 3 (a) and 16 (b) for flesh firmness and crispness retainability during cold storage in hybrid populations from *Malus asiatica* Nakai. ‘Zisai Pearl’ × *M. domestica* Borkh. ‘Red Fuji’. FFIII, FFII and FI represent the type III, type II and type I of flesh firmness retainability; FCIII, FCII and FCI indicate type III, type II and type I of flesh crispness retainability, respectively. The solid horizontal lines represent significant thresholds for QTLs on the maternal (red), paternal (blue) and both (black) parents. The position of candidate genes *MdERF3* (MD03G1194300) and *MdERF118* (MD16G1043500) were marked with red solid dots.
**Figure S6** Heatmaps showing differentially expressed genes of flesh firmness (a) and crispness (b) retainability detected by RNA‐seq of hybrids from *Malus asiatica* Nakai. ‘Zisai Pearl’ × *M. domestica* Borkh. ‘Red Fuji’. FFIII, FFII and FI represent type III, type II, and type I of flesh firmness changes; FCIII, FCII and FCI indicate type III, type II, and type I of flesh crispness changes, respectively. The numbers following the sample names indicate time points of sampling.
**Figure S7** Gene Ontology (GO) term enrichment (a, b and c) and KEGG pathway analysis (d, e and f) of the flesh firmness changes type III, type II and type I
**Figure S8** Gene Ontology (GO) term enrichment (a, b and c) and KEGG pathway analysis (d, e and f) of the flesh crispness changes type III, type II and type I

**Table S1** Phenotype segregation of initial flesh firmness/crispness at harvest and their retainability in hybrids ‘Zisai Pearl’ × ‘Red Fuji’, ‘Zisai Pearl’ × ‘Golden Delicious’ and ‘Jonathan’ × ‘Golden Delicious’ in year 2016–2017.
**Table S2** QTLs of apple fruit firmness/crispness retainability in hybrids of ‘Zisai Pearl’ × ‘Red Fuji’ identified by using BSA‐seq.
**Table S3** The fragments per kilobase of exon per million fragments mapped (FPKM) value of differentially expressed genes by RNA‐seq.
**Table S4** Differentially expressed genes of flesh firmness retainability in ethylene, cell wall degradation, ABA, GA, JA, and auxin synthesis and response pathways.
**Table S5** Differentially expressed genes of flesh crispness retainability in ethylene, cell wall degradation, ABA, GA, JA, and auxin synthesis and response pathways.
**Table S6** Candidate genes predicted from QTLs for apple flesh firmness and crispness retainability.
**Table S7** Kompetitive allele‐specific PCR (KASP) markers for apple flesh firmness and crispness retainability designed on candidate genes within QTL intervals.
**Table S8** The marker genotypes, observed phenotype values (OPV) and genomics‐predicted values (GPV) of apple flesh firmness retainability in hybrids randomly chosen from populations of 'Zisai Pearl' × 'Red Fuji', 'Zisai Pearl' × 'Golden Delicious', and 'Jonathan' × 'Golden Delicious'.
**Table S9** The marker genotype effect estimations of apple flesh firmness retainability in 473 hybrids randomly chosen from populations of 'Zisai Pearl' × 'Red Fuji', 'Zisai Pearl' × 'Golden Delicious', and 'Jonathan' × 'Golden Delicious'.
**Table S10** The marker genotypes, observed phenotype values (OPV) and genomics‐predicted values (GPV) of apple flesh crispness retainability in hybrids randomly chosen from populations of 'Zisai Pearl' × 'Red Fuji', 'Zisai Pearl' × 'Golden Delicious', and 'Jonathan' × 'Golden Delicious'.
**Table S11** The marker genotype effect estimations of apple flesh crispness retainability in 394 hybrids randomly chosen from populations of 'Zisai Pearl' × 'Red Fuji', 'Zisai Pearl' × 'Golden Delicious', and 'Jonathan' × 'Golden Delicious'.
**Table S12** Pearson's correlation coefficient (*r*) between observed phenotype values and genomics‐predicted values of apple flesh firmness retainability in fivefold cross‐validation.
**Table S13** Pearson's correlation coefficient (*r*) between observed phenotype values and genomics‐predicted values of apple flesh crispness retainability in fivefold cross‐validation.
**Table S14** Simulative selection by genomics‐assisted prediction model for apple flesh firmness retainability in 473 hybrids randomly chosen from ‘Zisai Pearl’ × ‘Red Fuji’, ‘Zisai Pearl’ × ‘Golden Delicious’ and ‘Jonathan’ × ‘Golden Delicious’.
**Table S15** Simulative selection by genomics‐assisted prediction model for apple flesh crispness retainability in 394 hybrids randomly chosen from ‘Zisai Pearl’ × ‘Red Fuji’, ‘Zisai Pearl’ × ‘Golden Delicious’ and ‘Jonathan’ × ‘Golden Delicious’.
**Table S16** The primer sequences used for qRT‐PCR, molecular interaction and gene cloning.
